# Enhancing offspring hypothalamic-pituitary-adrenal (HPA) regulation via systematic novelty exposure: the influence of maternal HPA function

**DOI:** 10.3389/fnbeh.2014.00204

**Published:** 2014-06-05

**Authors:** Sarah M. Dinces, Russell D. Romeo, Bruce S. McEwen, Akaysha C. Tang

**Affiliations:** ^1^Department of Psychology, University of New MexicoAlbuquerque, NM, USA; ^2^Neuroscience and Behavior Program, Department of Psychology, Barnard College of Columbia UniversityNew York, NY, USA; ^3^Laboratory of Neuroendocrinology, Rockefeller UniversityNew York, NY, USA; ^4^Department of Neurosciences, University of New MexicoAlbuquerque, NM, USA; ^5^Program in Cognitive Neuroscience, BCS/SBE, National Science FoundationArlington, VA, USA

**Keywords:** HPA, stress, maternal mediation, maternal modulation, early experience, enriched environment, neonatal handling, novelty

## Abstract

In the rat, repeated brief exposures to novelty early in life can induce long-lasting enhancements in adult cognitive, social, emotional, and neuroendocrine function. Family-to-family variations in these intervention effects on adult offspring are predicted by the mother’s ability to mount a rapid corticosterone (CORT) response to the onset of an acute stressor. Here, in Long-Evans rats, we investigated whether neonatal and adulthood novelty exposure, each individually and in combination, can enhance offspring hypothalamic-pituitary-adrenal (HPA) regulation. Using a 2 × 2 within-litter design, one half of each litter were exposed to a relatively novel non-home environment for 3-min (Neo_Novel) daily during infancy (PND 1–21) and the other half of the litter remained in the home cage (Neo_Home); we further exposed half of these two groups to early adulthood (PND 54–63) novelty exposure in an open field and the remaining siblings stayed in their home cages. Two aspects of HPA regulation were assessed: the ability to maintain a low level of resting CORT (CORTB) and the ability to mount a large rapid CORT response (CORTE) to the onset of an acute stressor. Assessment of adult offspring’s ability to regulate HPA regulation began at 370 days of age. We further investigated whether the novelty exposure effects on offspring HPA regulation are sensitive to the context of maternal HPA regulation by assessing maternal HPA regulation similarly beginning 7 days after her pups were weaned. We found that at the population level, rats receiving neonatal, but not early adulthood exposure or both, showed a greater rapid CORTE than their home-staying siblings. At the individual family level, these novelty effects are positively associated with maternal CORTE. These results suggest that early experience of novelty can enhance the offspring’s ability to mount a rapid response to environmental challenge and the success of such early life intervention is critically dependent upon the context of maternal HPA regulation.

## Introduction

Early life experiences can have powerful, long-lasting impacts on adult function (Bowlby, 1969; Ainsworth et al., 1979; Rutter, 1981). While a large body of literature deals with the negative consequences of early life stress on child development and its various animal models (Harlow et al., 1965; Bowlby, 1969; Rutter, 1981; Suomi, 1997; Bruce et al., 2013) relatively less is known regarding early life interventions that may bring about long lasting positive consequences in adult life. Building upon the earlier experimental paradigms of neonatal handling (Levine, 1960; Denenberg, 1964) and enriched environment (Rosenzweig, 1966; Weiler et al., 1995), we developed neonatal novelty exposure, an early life intervention that induced consistent and positive consequences across a wide range of functional domains, multiple levels of analysis, and multiple development stages (Tang and Zou, 2002; Tang et al., 2003b, 2006, 2008, 2012a,b, 2014; Akers et al., 2008).

The neonatal novelty exposure procedure captures one particular element shared between the neonatal handling and enriched environment paradigms, that is an increase in the novelty of the environment. In the case of the handling paradigm, the pups were taken away from the familiar home environment to a relatively novel non-home cage and in the case of enriched environment; the standard laboratory-rearing environment is augmented with not only more complex, but also changing stimuli. In both cases, the manipulation involves repeated environmental changes without which there would be no novelty. An organism’s response to novelty at behavioral and physiological levels are thought to contribute to individual differences in both physical and mental resilience or vulnerability to environmental challenge (Karatsoreos and McEwen, 2013).

To isolate novelty as a critical factor, we exposed rat pups to a relatively novel non-home cage as in the case of handling paradigm; but we controlled for other factors, such as maternal separation and experimenter handling, by using a within-litter instead of a between-litter design. Specifically, this entails having half of each litter spend a brief time (3 min daily) away from the familiarity of the home environment for the first 2–3 weeks of life, while the other half of the litter remains in the familiar home cages. With such a within-litter, or split-litter design, the offspring receiving and not receiving the novelty exposure treatment share the same mother; therefore, any intervention effect cannot be confounded by genetics.

Intervention-induced enhancement in spatial working and reference memory (Tang et al., 2011c), social recognition memory (Tang et al., 2003b), social dominance (Tang et al., 2006; Akers et al., 2008), and habituation to novelty (Tang et al., 2006) are detectable at least in late adulthood if not during senescence. Combining this intervention with measurement of maternal characteristics, we found that variations in the intervention effect across different rat families can be explained by specific maternal characteristics (Akers et al., 2008; Tang et al., 2011a,c, 2012a,b; Reeb-Sutherland and Tang, 2012). One particular maternal characteristic is her ability to mount a rapid corticosterone (CORT) response to environmental challenge. This ability has been operationalized by a measure of CORT concentration in the blood sample obtained 5 min after the onset of a 1 min swim stressor and this operational definition has been effective in predicting multiple offspring outcomes (Tang et al., 2011a, 2012a,b).

Converging evidence suggest that these intervention effects may arise in part from an underlying intervention effect on the developing offspring’s hypothalamic-pituitary-adrenal (HPA) function, specifically the offspring’s ability to regulate its own CORT output. Novelty can lead to an increase in circulating CORT in 2 day old rat pups (Denenberg et al., 1967). Maternally generated CORT can also provide input to the pups’ developing HPA axis because mother’s CORT can be provided to the pups via her milk (Angelucci et al., 1983). Such stimulation of the HPA axis via either the maternally generated or infant generated CORT is likely to produce changes in HPA function relative to rats that experience little or no input to their HPA axis. Indirect measures of HPA function from the adult offspring showed that novelty-exposed rats have more functioning GR receptors (Zou et al., 2001), show greater plasticity in circulating CORT after repeated social competition (Akers et al., 2008), and have a lower resting level of CORT (Tang et al., 2003b).

As the majority of the early experience studies focused on the delayed peak hormonal response and recovery after the peak response (Levine, 1960; Meaney et al., 1988), here we present a study that specifically investigates a relatively understudied aspect of HPA function in both dams and their adult offspring—the ability of the HPA axis to generate a rapid increase in its output as an initial response to environmental challenge. This ability may be of particular importance to rapid learning and adaptation when the animal is facing novelty or unexpected or sudden changes in its environment, particularly given CORT’s action on the mineralocorticoid receptors (MRs) and its facilitative effect on synaptic plasticity (Joëls et al., 2012). To better focus on this initial rise, we measured CORT from blood samples collected after a very short delay of 5 min from the onset of a very brief acute stressor (1 min swim).

This study has the following specific aims: first, investigating whether neonatal and early adulthood novelty exposure each alone and in combination can produce a positive intervention effect on the adult offspring’s HPA regulation, i.e., an enhancement in HPA regulation; second investigating whether any family-specific intervention effects on offspring HPA function are sensitive to the context of maternal HPA regulation. Methodologically, the present study seeks to expand the range of indices for HPA regulation by providing further evidence that the initial CORT response is an effective index of HPA regulation and can be used as a biomarker, capable of making long-range family-specific predictions for long-lasting early experience effects on offspring HPA regulation.

## Materials and methods

### Animals

Long Evans male offspring (*N* = 94) from 19 hooded dams participated in the present study. The dams (Charles River, Portage, MI) arrived at the vivarium 12 days before giving birth and were approximately 3 months of age. Litter size at birth ranged from 9 to 16 and within 8 h after birth litters were culled to 8 pups maximizing the number of male pups. Pups were weaned on postnatal day (PND) 21 and were housed individually in transparent plastic cages (51 × 25 × 22 cm) with a 12 h light/dark cycle (lights on at 8:00 a.m.) and food and water ad libitum. All experimental procedures were in accordance with the Institutional Animal Care and Use Committee at the University of New Mexico. All of the experimental procedures, with the exception of the timing of the HPA measures, have been described in detail in previous publications (Tang et al., 2006, 2011a,b,c, 2012a,b; Akers et al., 2008; Yang and Tang, 2011).

### Experimental design and procedures

**Figure 1:**
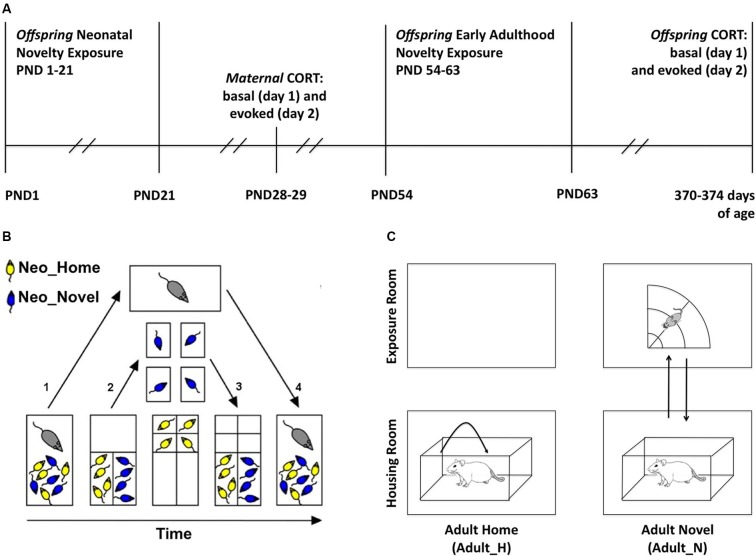
**Experimental methods**. **(A)** Timeline. PND: postnatal day. **(B)** Sequential steps in the neonatal novelty exposure procedure (split-litter design, PND 1–21): after the assignment of pups to neonatal novelty exposure conditions (NeoN, NeoH), the following is performed daily. (1) Dam is removed from the home cage; (2) Novel and Home pups (NeoN, NeoH) are sorted into the two small compartments, Novel pups are transferred to individual non-home cages and Home pups are transferred to individual compartment in home cage, both receiving the same amount of experimenter touch; (3) After 3 min, Novel and Home pups were both returned to the home cage; and (4) After all dividers are removed, dam is returned to the home cage. **(C)** Early Adulthood novelty exposure (PND 54–63): the following is performed daily. Right: the Adult_N rats (Neo_H-Adult_N = HN; Neo_N-Adult_N = NN) were transported to the exposure room and placed in the open field for 3 min; Left: Adult_H rats (Neo_H-Adult_H = HH; Neo_N-Adult_H= NH) were picked up twice and replaced back into their home cage in the housing room.

To study whether repeated and brief neonatal and early adulthood novelty experience can induce long lasting changes in the offspring’s HPA function and how maternal individual differences in her regulation of her own HPA might affect offspring HPA function, we first exposed the offspring to neonatal and adulthood novelty exposure procedures. We then obtained measures of maternal self-stress regulation 7 days after the pups were weaned and we measured offspring HPA regulation in late adulthood as shown in the experimental timeline (Figure 1A). A 2 × 2 within-litter factorial design was used in which siblings within each rat family were divided and assigned to four experimental conditions defined by whether the rat received exposures to a relatively novel non-home environment (N) or stayed only within the home cage (H) during neonatal (Neo) development or early adulthood (Adult) (Group 1: Neo_H-Adult_H (HH); Group 2: Neo_H-Adult_N (HN); Group 3: Neo_N-Adult_H (NH); Group 4: Neo_N-Adult_N (NN)). While many rodent studies of early life environmental effects use individual rats as units of analysis, we used litters as our units of analysis to both increase statistical power and avoid treating multiple animals from a given litter as independent from one another. The latter can potentially exaggerate the statistical significance.

### Neonatal novelty exposure

Neonatal novelty exposure was conducted from PND 1–21 in the housing room (Figure 1A). The pups were identified by the experimenter as either Neo_N or Neo_H rats and sorted into separate compartments (Figure 1B) within the home cage. The neonatal novelty exposure procedure involved taking a subset of pups away from the familiarity of their home cage for 3 min daily (Neo_N) while their matched control siblings (Neo_H) remained in the home cage (Figure 1B). With this split-litter or within-litter design, the novelty exposure effect is isolated from the effects of experimenter handling, maternal separation of the pups, and maternal stress (Tang, 2001; Tang et al., 2003b, 2006, 2011c, 2012a).

This novelty exposure procedure is a result of further improvement from that used in previous studies (Tang, 2001; Tang et al., 2003a, 2006) with an added control of littermate separation (see details below and Figure 1B). On PND 0 (postnatal day), group membership was indicated by tattooing the hind paws. Both hind paws were marked to avoid a differential stimulation effect on the left and right brain given the various known effects of early stimulation on functional brain asymmetry (Tang, 2003). Two patterns were used: with markings on the 1st digit of the left paw and 5th digit of the right paw (L1R5) or vice versa (L5R1). To avoid the novelty exposure treatment from being confounded by the patterns of marking, we marked half of the Novel rats with L1R5 and the other half with L5R1 and marked the Home rats similarly.

The Neo_N pups were individually transferred into separate small non-home cages (30 × 19 × 13 cm) lined with fresh bedding similar to the bedding used in the home cage and the Neo_H pups were also individually transferred into separate small compartments within the home cage. The timing of transfer for each Neo_N pup was matched to that of the Neo_H pup to maximize the similarity in experience. Both Neo_H and Neo_N pups remained isolated from their littermates for 3 min. To ensure that the duration of pup–dam separation is the same for the Neo_H and Neo_N rats, the dams were only returned to the home cage after all of her pups were returned to the home cage.

These steps ensured that experimenter–pup, pup–pup, and dam–pup interactions were matched between Neo_N and Neo_H pups. Note that the total duration of separation between dams and all of her pups, including time spent on sorting the Novel and Home pups into separate compartments (Figure 1B), 3 min spent in individual cages or compartments, transfer times between these locations, was no more than 15 min, which does not constitute the kind of maternal separation known to result in detrimental effects (Sánchez et al., 2001).

### Early adulthood novelty exposure

During PND 54–63 (Figure 1A; early adulthood), half of the neonatal novelty-exposed (Neo_N) and half of the home-staying (Neo_H) rats in each litter were exposed to an additional novelty experience (Adult_N) in a sectored open field (radius: 75 cm) for 3 min daily while the other half remained at home (Adult_H). This novelty exposure was carried out daily between 12:00 and 17:00 h. The early adulthood novelty exposure procedure was carried out in the animal housing room and was meant to be analogous to the neonatal novelty exposure in that both involved giving a subset of the animals exposure to a brief repeated stressor; however, these procedures were not intended to be identical nor could it be expected to have identical effects.

Within each litter, Adult_H rats were handled within the housing room first, followed by exposing the Adult_N rats to the sectored open field. The order of Neo_N and Neo_H rats was counterbalanced within the Adult_H and Adult_N conditions. If a rat was assigned to the Adult_N condition (HN or NN), it was first removed from the housing shelf to the transporting cart to be subsequently transported to a separate room for novelty exposure. Up to four rats were transported together and individually placed and remained in one of the four separate sectors of a circular open field for 3 min before being returned to the housing room (Figure 1C). If a rat was assigned to the Adult_H condition (HH or NH), it was also removed from the housing shelf to the transporting cart where it was twice picked up and replaced to the other end of their own home cage to match the amount of handling to be experienced by the Adult_N rats during their exposure to the sectored open field. They were directly returned to the housing shelf after this handling.

### Maternal and offspring individual differences in HPA function

Shortly after weaning, maternal resting corticosterone (CORT_B_) and post-swim circulating CORT (CORT_S_) levels were measured from blood samples collected, via tail nicking, on PND 28 and PND 29, 7–8 days after weaning. This specific delay from weaning was selected based on a balance among the following constraints: that they should not be (1) too distant temporally from the pups’ weaning to fail capturing the pups’ pre-weaning maternal environment; (2) too close to weaning as the disturbances associated with weaning may potentially affect the maternal CORT levels measured; and (3) measured during nursing to avoid further stressing the dams due to blood sampling. The timing of the maternal blood sample collection was set between 1–3:30 pm at the nadir of the circadian cycle to capture the “true” baseline within the full dynamic range of the individual’s own circulating CORT. Similarly defined CORT measures were collected from the offspring starting when the rats were 370 days of age. The timing of the sample collection was centered around the nadir of the circadian cycle but during a larger time window (10 am-4:30 pm). This is a compromise made in order to minimize variations due to testing on multiple days.

We matched the time of day for collection of CORT_B_ and CORT_S_ to ensure that the basal and evoked measures were obtained at the same time of day for each given rat. For CORT_B_ samples, rats were transported from the housing room directly to the blood collection room with no more than a 3 min delay from the time the rat was removed from the housing room. For CORT_S_ samples, rats were transported from the swim test room to the blood collection room 5 min after the onset of the 1 min swim test (water temperature of ~21°C). An evoked CORT response, CORT_E_, was defined as the difference between CORT_S_ and CORT_B_ normalized by CORT_B_ (CORT_E_ = (CORT_S_ − CORT_B_)/CORT_B_ × 100).

All samples were processed in a single assay in duplicate. Each sample, containing ~200 µL of blood, was centrifuged, and the plasma was extracted and then stored at −20°C until radioimmunoassay (RIA) was performed. Plasma CORT concentration was measured using the Coat-a-Count CORT Kit (Diagnostic Products, Los Angeles, CA). The lower limit of detection for the maternal CORT samples was 10.1 ng/mL and the intra-assay coefficient of variation was 4.8%. The offspring samples had a lower limit of detectability at 9.5 ng/ml, and the intra-assay coefficient of variation at 5.4%. It should be noted that we were unable to obtain a sufficient amount of plasma from some rats. Hence, the number of samples for the evoked CORT measure (*Ns* = 11 and 12 for the NH and HN) was smaller than the number of samples for the basal CORT measure (*Ns* = 14, 13 for the NH and HN). This is likely due to increased constriction of blood flow in the tail after exposure to the cold water of the swim stressor.

**Table:** 

	***Dams***				***Offspring***				
	**N**	**Min**	**Max**	**Mean ± SEM**	**N (litters)**	**n (offspring)**	**Min**	**Max**	**Mean ± SEM**
CORT_B_ (ng/mL)	17	20.6	217.1	103.6 ± 18.3	18	89	14.4	240.2	101.9 ± 5.7
CORT_S_ (ng/mL)	16	176.1	539.1	345.9 ± 29.4	18	91	96.0	377.2	219.5 ± 7.0
CORT_E_ (%CORT_B_)	16	28.7	860.6	399.4 ± 73.6	18	82	−43.7	425.9	137.2 ± 12.2

### Data analysis

Two-way analyses of covariance (ANCOVAs) were performed with litters as units of analysis and neonatal and early adulthood novelty exposure as two within factors. Maternal basal and evoked CORT were used separately as covariates (CORT_MB_ and CORT_ME_), and offspring basal and evoked CORT were used separately as the dependent measures (CORT_OB_ and CORT_OE_). Three specific hypothesis-driven one-way ANCOVAs were performed to test the following specific hypotheses: (1) neonatal novelty exposure enhances offspring HPA regulation and this enhancement is modulated by maternal HPA regulation (NH vs. HH: one-tailed); (2) early adulthood novelty exposure enhances offspring HPA regulation (HN vs. HH: one-tailed) and this enhancement is modulated by maternal HPA regulation; and (3) combined novelty exposure affects offspring HPA regulation (NN vs. HH: two-tailed) and its modulation by maternal HPA regulation. As the two-way ANCOVAs did not reach statistical significance (*ps* > 0.163), only one-way ANCOVA results, along with descriptive statistics on the raw CORT measures are reported in the Results section. Furthermore, as no statistically significant combined novelty exposure effects were found for any of the offspring CORT measures from the one-way ANCOVA, only the Neonatal and Adulthood Novelty Exposure Effects are discussed in the Results section.

Novelty exposure effects for *each litter* are indexed by the following three novelty scores (NE scores): the Neonatal Novelty Exposure Score (NH - HH), the Early Adulthood Novelty Exposure score (HN - HH), and the Joint Neonatal and Adulthood Novelty Exposure Score (NN - HH). These scores quantify the differences between the novelty-exposed and the home-staying animals indicating a litter specific change induced by the respective novelty exposure treatment. For example, a larger positive NE score for CORT_E_ indicates a novelty exposure induced increase in CORT_E_. Based on previous novelty exposure studies that showed novelty-induced *enhancement* in HPA regulation (Zou et al., 2001; Tang et al., 2003b; Akers et al., 2008), the hypotheses for the main Novelty Effects from the first two one-way ANCOVAs are directional, therefore, the test of main effects in these two ANCOVAs are one-tailed. For the ANCOVA on the Joint Novelty Exposure Effect, because no prior theoretical prediction can be made, the test is non-directional, therefore two-tailed.

Data were first checked for violation of assumptions of normality and heterogeneity of variance and appropriate transformations of data were performed where needed. Using bag-plots (Rousseeuw et al., 1999; Wolf, 2007), outliers in the raw data were detected and various transformations were examined (including square roots, replacement with the next most extreme value, replacement with the average value, and deletion of outliers) to determine the appropriate transformation for the final analysis. Along with the report of *F*- and *p*-values, effect sizes are also reported (effect size f: small: < 0.1, medium: 0.1 < *f* < 0.25; large: 0.25 < *f* < 0.40) (Rosenthal and Rosnow, 1991).

## Results

Descriptive statistics for the resting CORT (CORT_B_) obtained on Day 1 of blood sample collection, post-swim CORT (CORT_S_) obtained on Day 2 of blood sample collection, and the rapid CORT response to the 1-min swim stressor, computed as a percent increase from the baseline (CORT_E_) are presented separately for the dams and offspring with the individual rat as unit of analysis. Descriptive statistics for the dams (left) have different *Ns* (16, 17) because some litters have missing values for either maternal CORT_B_ or CORT_S_. Descriptive statistics for the offspring (right) are computed from more litters (*Ns* = 18) as some litters may have the maternal CORT measure as missing values but nevertheless have offspring CORT measures. Notice that the *Ns* and *ns* for CORT_E_ are smaller than the *Ns* and *ns* for CORT_B_ and CORT_S_ because a missing value of either CORT_B_ or CORT_S_ will result in the loss of CORT_E_. The negative value of minimum CORT_E_ for the offspring is possible because CORT_B_ and CORT_S_ are based on blood samples collected on separate days. As these raw data contained outliers, the following ANCOVA results were obtained on appropriately transformed data to meet the assumptions of ANCOVA.

### Neonatal novelty exposure increased adult offspring CORT_E_

**Figure 2:**
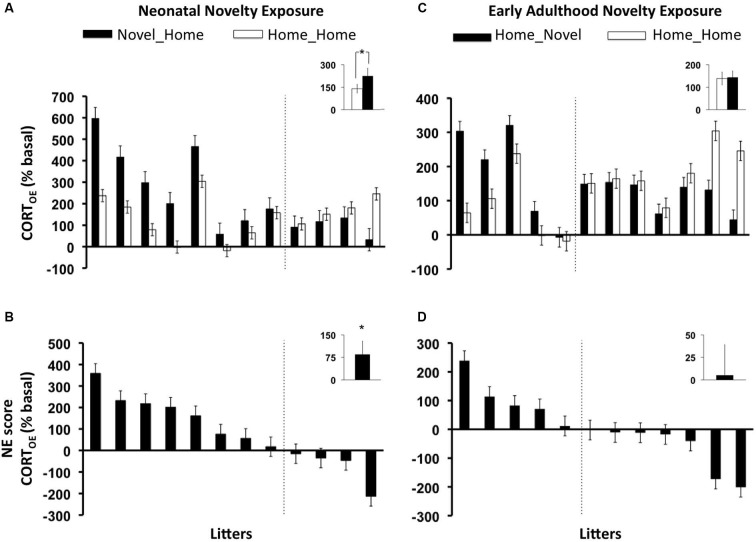
**Litter-by-litter variations and group averages in effect of novelty exposure on offspring rapid evoked CORT response to 1 min swim stressor (CORT_OE_) during infancy (AB) and early adulthood (CD)**. Novel_Home: animals who experienced *neonatal* novelty exposure alone, Home_Novel: animals who experienced *early adulthood* novelty exposure alone, Home_Home: animals who experienced no novelty exposure during either period (controls). **AC**: litter by litter differences between the Novel and Home siblings. **BD**: litter-by-litter Novelty effect score (NE score) = (Litter_AVG_NOVEL_- Litter_AVG_HOME_). **AB**: neonatal novelty exposure significantly increased evoked CORT measure in the offspring (CORT_OE_), **CD**: no statistically significant effects were found for early adulthood novelty exposure. Note that the orders of the litters in all panels are based on the NE scores. Therefore, the position on the “litter” axis does not correspond to the same litter.

As predicted, a significant main Neonatal Novelty Exposure Effect (NH vs. HH) on CORT_OE_, was revealed by a one-way ANCOVA, with the novel rats showing a greater rapid CORT response (CORT_E_) than the home rats (Figures 2A,B) (*F*_(1, 11)_ = 3.41, *p* = 0.0475 one-tailed, *f* = 0.588). No significant results were found for those offspring who were exposed to early adulthood novelty (HN) (Figures 2C,D) (CORT_OE_: *F*_(1,11)_ = 0.037, *p* = 0.425 one-tailed, *f* = 0.055). The litter-specific, novelty-induced, enhancement for each rat family was indexed by a litter-based novelty effect score (NE score) defined as Litter_AVG_NOVEL_ − Litter_AVG_HOME_, shown in Figures 2B,D. The *neonatal* novelty exposed siblings’ evoked CORT response was 75% higher than their Home siblings relatively to their baseline (Figure 2B, inset). In comparison, the *early adulthood* novelty exposed animals only showed approximately a 6.5% difference (Figure 2D, inset). These results show that repeated brief neonatal but not early adulthood novelty exposure, lead to enhancement in the offspring’s ability to mount a rapid response to environmental challenge, and that this enhancement is present in mid-adulthood (13 months). We further examined how novelty exposure affected *individual* rat families. There exists a large bidirectional range of novelty exposure effects: some rat families show novelty-induced enhancement (positive NE scores) and others show an impairment effect (negative NE scores) (Figures 2B,D). These observations highlight the obvious, yet largely neglected, fact that a given early-life intervention may produce positive effects on average, but opposite effects may be observed for a subset of individuals.

### Maternal modulation of neonatal novelty exposure effect on adult offspring CORT_E_

**Figure 3:**
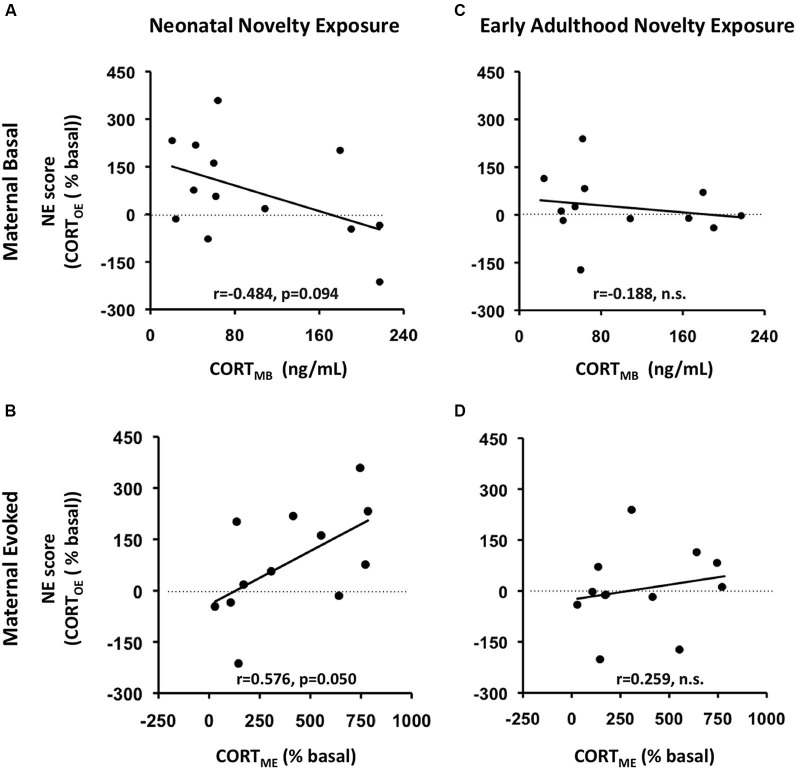
**Maternal rapid evoked CORT response (CORT_ME_) but not basal CORT (CORT_MB_) predicts litter-specific novelty effect, measured by NE score, defined as Litter_AVG_NOVEL_- Litter_AVG_HOME_)**. **AB**: prediction of *neonatal* novelty exposure effects; **CD**: prediction of *early adulthood* novelty exposure effects. **AC**: prediction by maternal basal CORT (CORT_MB_); **BD**: prediction by maternal rapid evoked CORT response (CORT_ME_). **(A)** CORT_MB_ marginally predicts novelty effect of offspring exposed to neonatal novelty. **(B)** The greater the maternal evoked CORT response (CORT_ME_), the greater the novelty-induced enhancement in offspring evoked CORT (CORT_OE_).

To relate this bidirectional variation to the context of maternal individual differences in self-stress regulation, we computed correlations between the NE scores and the maternal basal and evoked CORT measures (CORT_MB_, CORT_ME_ respectively) in addition to *F*-statistics from ANCOVAs for the interaction effects between Neonatal Novelty Exposure and Maternal CORT measures. We found that the direction and magnitude of the novelty exposure effect was indeed correlated with the measure of maternal CORT_E_, denoted as CORT_ME_ (Figure 3B) (Correlation: *r* = 0.553, *p* = 0.05, ANCOVA interaction effect: *F*_(1,11)_ = 5.078, *p* = 0. 048, *f* = 0.713). Specifically, enhancement of offspring CORT_E_, denoted as CORT_OE_, is observed in the context of high CORT_ME_, while a lack of such enhancement, or a small impairment, was observed in the context of low CORT_ME_. Only a marginally significant effect was found by the ANCOVA with CORT_MB_ as the covariate (Figure 3A) (CORT_OE_: *r* = −0.420, *p* = 0.135, *F*_(1,12)_ = 3.356, *p* = 0.094, effect size *f* = 0.553). Note that the effect size, *f, is large*. No significant effects were found from the ANCOVAs on early adulthood novelty exposure effects (Figures 3C,D). There were no significant effects found from ANCOVAs involving maternal or offspring basal CORT. This may be partially explained by the method used to collect the basal CORT measure, as rats were removed from the housing room one at a time, which may have increased the CORT levels of those animals sampled later due to potential disturbances in the vivarium.

## Discussion

In a longitudinal study of rodent families consisting of dams and their adult male offspring, we found that rats who experienced a daily 3 min exposure to a relatively novel non-home environment during the first 3 weeks of life differed significantly in their rapid CORT response to the onset of an acute stressor from their control littermates who stayed only in the familiar environment of the home cage. Specifically, at mid-adulthood (370 days of age), Novel offspring showed a greater rapid evoked CORT response to the onset of a 1-min swim stressor than their Home siblings.

The rat families differed in the magnitude and direction of this *litter-based* novelty exposure effect, defined as the difference score between the average of all the Novel littermates and the average of all the Home littermates within a given litter. Furthermore, this litter-specific novelty effect is positively correlated with the mother’s rapid evoked response—the greater a rapid response the mom can mount, the greater the novelty-induced enhancement was found in her offspring. This finding suggests that family-to-family variations in the novelty effect may be modulated by maternal self-regulation of her own HPA axis.

It is interesting to note that the correlation between the maternal CORT measures and novelty effects on offspring was statistically significant for the evoked CORT response (CORT_E_) but only marginally significant for the resting CORT (CORT_B_). As the neuroendocrine mechanism determining the maintenance of the basal circulating CORT level and the mechanism determining how fast the animal can mount a CORT response are likely to involve different aspects of the HPA axis, it is not entirely surprising that only one of the two CORT measures have reached statistical significance in its predictive power for the novelty effect. It is also possible that this differential predictive power results in part from the difference in the sensitivity or variance of the measures themselves.

In contrast to these *neonatal* (PND 1–21) effects, we did not observe a significant effect from *early adulthood* novel exposure (PND 54–63). It is important to note that these contrasting findings do not necessarily imply that the neonatal intervention is more effective than the early adulthood intervention, or that the first 3 weeks of life constitutes a critical period. The reasons behind this caution in interpretation is that the procedures for these two interventions are not identical, and even if they were procedurally identical, their effects on the offspring cannot be expected to be identical because animals of different ages would have had different cumulative experiences, thus different response at the time of intervention.

Due to the scope of the study, the offspring investigated in the present study were males. One should not assume that the same outcome would hold for the female offspring because there are known differences in HPA function between the two sexes (Bangasser and Valentino, 2012; Handa and Weiser, 2014) and the effects of novelty exposure on several behavioral measures also differed between the male and female offspring (Tang et al., 2003a; Akers et al., 2004; Reeb and Tang, 2005).

### Conceptual meaning of CORT_E_

Behind the CORT_E_ measure used in the present study is a very simple rationale based upon the adaptive value of the acute HPA response to an acute challenge for an animal in its daily life. The rapid and efficient turning on of a CORT as well as an adrenalin response to a stressor determines rapid and efficient trafficking of immune cells to organs and tissues where they can defend against a pathogen or repair a wound (Dhabhar and McEwen, 1999; Dhabhar et al., 2012). Moreover, acute elevation of CORT, as well as adrenalin to a stressor in a novel environment, enhances contextual memory that would be useful to avoid danger in the future (Cahill et al., 1994; Okuda et al., 2004; Roozendaal et al., 2006). Finally, a timed, acute CORT elevation protects against the development of delayed PTSD-like fear along with increased spine synapses in basolateral amygdala (Zohar et al., 2011; Rao et al., 2012). Indeed, the CORT_E_ measure has served as an effective biological marker for other behavioral and endocrine measures within the same individual (Tang et al., 2011a, 2012a,b), as well as from one generation to another (Tang et al., 2011a, 2012a,b).

It is also important to make clear the distinctions between the conceptual meanings captured by the present operational definition and those captured by other frequently used HPA regulation measures in the literature (Golden et al., 2011). Specifically, the CORT_E_ value in the present study is conceptually and operationally different from the high cortisol measures used in some studies of children, in which samples were collected during school hours and the corresponding cortisol levels. Such cortisol measures do not reflect responses evoked by explicit and discrete events (Lupien et al., 2000). The high CORT_E_ value in the present study also differs from the “high CORT” level observed a couple of hours after prolonged restraint (≥20 min) (Meaney et al., 1988) which reflects the rate of return to baseline instead of the rising rate of CORT output.

### Causes of early experience effects

The present study offers new evidence for a possible causal link between early experience of novelty and the ability of adults to mount a rapid CORT response to environmental challenge, providing converging evidence along with other previously reported positive effects of early life experience on the function of the HPA axis from studies using a diverse range of paradigms (Levine, 1971; Champagne et al., 2003; Parker et al., 2006; Catalani et al., 2011; Simpson and Kelly, 2011). Unique to the present study is its improved ability to pinpoint what specific factors cause the observed differences in offspring HPA function. As Novel and Home rats were randomly assigned to their respective conditions and differed only in the 3 min experience of a relatively novel non-home environment, with maternal separation, maternal genetics, maternal stress experience, and experimenter handling all matched, we are able to conclude that the observed difference in offspring CORT_E_ is most likely caused by the brief repeated experience of novelty.

The present study also offers new evidence for maternal circulating stress hormone as a critical dimension of the complex developmental context, enabling the same early life intervention to produce bidirectional effects. It provides converging evidence for the maternal modulation hypothesis, which states that the direction and amplitude of an early life intervention effect on the developing individual is modulated by its maternal context (Tang et al., 2014). This conclusion is also supported by several earlier findings that manipulations of or variations in maternal circulating CORT lead to changes in offspring HPA function in both the animal model (Kalin et al., 1998; Catalani et al., 2011; Macrì et al., 2011; Tang et al., 2011a, 2012a,b) and humans (Papp et al., 2009; Laurent et al., 2012; Williams et al., 2013).

### Neural mechanisms underlying the novelty effect

What is the essence of the novelty exposure manipulation that leads to the observed difference in offspring HPA regulation? We consider the possibility that the essence of repeated brief novelty exposure is the repeated brief exposure of the developing brain to transient CORT increases. This possibility is strongly suggested by Catalani and colleagues’ finding that manipulating the mother’s milk CORT content can produce long-lasting changes in the offspring’s CORT stress response curve and GR receptor function (Catalani et al., 2011), by Denenberg and colleagues early finding that the action of taking the pups away from the homecage, and by Lyons and colleagues’ finding that intermittent exposure to a novel environment (referred to by the authors as intermittent stress treatment) can also produce some of the similar effects on HPA function (Parker et al., 2006). We believe that these early exposures to repeated, transient and moderate increase in circulating CORT resulted in long-lasting changes in HPA regulation and these changes have multiple expressions within different functional contexts.

Some of the most commonly explored expressions of altered HPA function are changes in GRs and MRs within the hippocampus. In *in vitro* brain slice physiology experiments (Zou et al., 2001), the inhibition of amplitude of population spikes recorded in the CA1 of the hippocampus by stress-like levels of CORT was significantly greater in the brain slices from the Novel rats than from the Home rats, suggesting a novelty effect on the availability of functioning GRs. These GR differences may contribute to a different cumulative history of CORT exposure (Sapolsky, 1992). Furthermore, in the same study, within minutes of the onset of CORT perfusion, a transient and small increasing trend in the population spike amplitude was observed only in brain slices from the Novel rats, possibly reflecting a novelty-induced increase in functionally available MR receptors. As the MRs are known to contribute to the initial phase of the stress response (Joëls et al., 2008, 2012), an increase in functionally available MRs may contribute to the greater ability of the Novel rats to mount an initial rapid increase in CORT output, here expressed as a greater CORT_E_ than the Home rats.

## Conclusions

In the present study, we provided evidence for a long-lasting positive effect of repeated and brief exposures to novelty on adult HPA regulation and for a dependence of this intervention effect on the specific maternal stress regulation context. This line of evidence reveals a critical yet poorly understood role played by the temporal dynamics of circulating CORT during early development and an urgent need in designing and implementing family specific interventions to create positive developmental outcomes. This study also provided further evidence for the effectiveness of a rapid CORT response measure as a maternal predictor for long-lasting early intervention effect on offspring development. These effective interventions combined with sensitive maternal and offspring functional measures may provide the needed insight into not only malfunction but also effective adaptation to challenges in the environment.

## Authors and contributions

Sarah M. Dinces: statistical analysis, interpretation of data, revising of the manuscript; Russell D. Romeo: CORT assay, interpretation of data; revising of the manuscript; Bruce S. McEwen: interpretation of data, revising of the manuscript; Akaysha C. Tang: conception and design of the study, drafting of the manuscript, data acquisition, statistical analysis, and interpretation of data.

## Conflict of interest statement

The authors declare that the research was conducted in the absence of any commercial or financial relationships that could be construed as a potential conflict of interest.
